# The role of immune-inflammatory markers in children with complicated and uncomplicated malaria in Enugu, Nigeria

**DOI:** 10.1186/s12865-024-00642-y

**Published:** 2024-07-23

**Authors:** Angela Ogechukwu Ugwu, Rebecca Chinyelu Chukwuanukwu, Friday Alfred Ehiaghe, Emmanuel Onyebuchi Ugwu

**Affiliations:** 1 Department of Haematology and Immunology, Faculty of Basic Clinical Sciences, College of Medicine, University of Nigeria Nsukka, Enugu, Enugu State 400001 Nigeria; 2 Department of Medical Laboratory Science, Faculty of Health Sciences and Technology, College of Health Sciences, Nnamdi Azikiwe University Nnewi Campus, Nnewi, Anambra State 420001 Nigeria; 3 Department of Obstetrics and Gynaecology, Faculty of Clinical Sciences, College of Medicine, University of Nigeria Nsukka, Enugu, Enugu state 40001 Nigeria

**Keywords:** Complicated malaria, Uncomplicated malaria, Cytokines, Immune cell ratios

## Abstract

**Background:**

There is currently insufficient data regarding immune parameters and relationship with severity of malaria infection in Enugu, Nigeria where the economic and social costs of the disease and its management are extremely high. This study was conducted to determine the relationship between malaria severity and some immune-inflammatory markers among malaria-infected children in Enugu, Nigeria.

**Methods:**

The study adopted a case control design. Eligible children were categorized into three groups — complicated, uncomplicated and healthy children. Pro-inflammatory cytokines –interferon-gamma (IFN-γ) and tumor necrosis factor-alpha (TNF-α); and anti-inflammatory cytokine — interleukin-10 (IL-10) were assayed using enzyme-linked immunosorbent assay (ELISA) technique, while immune cell ratios — neutrophil lymphocyte ratio (NLR) and monocyte lymphocyte ratio (MLR) were calculated from full blood count results.

**Results:**

The overall mean age of the participants was 7.3 ± 3.4 (range: 6 months − 12 years) and the male-female ratio was 1:1. There was no significant difference between the ages of the three groups (*P* = 0.44). The Mean levels of IFN-γ, TNF-α, and NLR were higher in complicated than uncomplicated malaria (266.9 ± 66.3pg/ml vs. 62.5 ± 6.4pg/ml, *p* < 0.001; 140.3 ± 30.0pg/ml vs. 42.0 ± 9.0pg/ml, *p* < 0.001; and 32.9 ± 16.2pg/ml vs. 17.8 ± 6.0pg/ml, *p* < 0.001, respectively); and higher in uncomplicated malaria than healthy children (62.5 ± 6.4pg/ml vs. 40.6 ± 9.1pg/ml, *p* < 0.001; 42.0 ± 9.0pg/ml vs. 105.7 ± 32.1, *p* < 0.001; 17.8 ± 6.0pg/ml vs. 18.7 ± 6.2pg/ml, *p* < 0.001, respectively). On the other hand, the mean level of IL-10 is higher in uncomplicated than complicated malaria (105.73 ± 32.06pg/ml vs. 40.60 ± 9.11pg/ml, *p* < 0.001). There was a positive correlation between NLR and IFN-γ (*r* = 0.815; *p* = 0.003), as well as NLR and TNF-α (*r* = 0.745; *p* = 0.002).

**Conclusion:**

Complicated malaria is associated with higher levels of pro-inflammatory cytokines while uncomplicated malaria is associated with higher levels of anti-inflammatory cytokines. NLR correlates positively with pro-inflammatory cytokines, and could be useful in evaluation for the severity of malaria infection.

## Introduction

Malaria accounted for 619,000 deaths in 2021, and the WHO (World Health Organization) African Region carries a disproportionately high share of the global malaria burden [1]. More than half of this global malaria burden is caused by *Plasmodium falciparum* in Sub Saharan Africa. Nigeria, is estimated to contribute approximately a quarter (25%) of global malaria burden. Children under 5 accounted for about 80% of all malaria deaths in the WHO African Region [2].

According to Center for disease control and prevention (CDC in 2022), malaria is classified as complicated and uncomplicated malaria [3]. The complicated malaria is typified with hyperparasitaemia of > 10%, vital organ derangement, or haematological or metabolic abnormalities [3, 4]. The effect on vital organs may result in shock, pulmonary edema, significant bleeding, acute renal injury, seizures, impaired consciousness and coma [3, 4]. Haematological or metabolic abnormalities may include but not limited to severe anaemia (haematocrit < 15%), jaundice (total bilirubin > 50 µmol/L with a parasite density > 100,000/uL as well), a plasma bicarbonate of < 15 mmol/L, lactate > 5 mmol/L and arterial pH < 7.25 [3, 4]. On the other hand, uncomplicated malaria consists of symptomatic *Plasmodium falciparum* infection with a positive parasitological test and parasitemia ≤ 5%, in the absence of symptoms consistent with severe malaria [3]. The uncomplicated malarial symptoms include general malaise, fever chills, headaches, nausea and vomiting [3, 4].

The elevated levels of pro-inflammatory cytokines and chemokines in peripheral blood are likely implicated in the immunopathology that manifests during the course of the disease [5, 6]. Also, the clearance of *Plasmodium falciparum* from the body depends largely on the cellular and humoral components of the adaptive immune system, both of which are heavily dependent on the CD4 + cells [7]. In the acute phase of *Plasmodium* infection, pro-inflammatory cytokines including tumour necrosis alpha (TNF-α), interferon gamma (IFN- γ), interleukin-6 (IL-6), and interleukin-8 (IL-8) have been reported to be produced in large quantities by the lymphocytes (CD4 + cells) [5]. The anti-inflammatory cytokines including interleukin-4 (IL-4) and interleukin-10 (IL-10) have also been reported to be produced by these cells in patients with *Plasmodium* infection [5]. These cytokines (pro-inflammatory and anti-inflammatory) are thus essential for regulating the *Plasmodium* parasite’s growth and clearance.

The capacity to control these pro-inflammatory reactions, presumably through processes of immunologic tolerance, may be a determinant of clinical immunity to malaria [8].

Despite the increasing knowledge of genetics and the molecular basis of *P. falciparum*, what determines the outcome of individual infections or how outcome varies with exposure at a population level remains largely unknown. A clear elucidation of the role of cytokines in the immune regulation of malaria parasite severity is essential. Understanding the relationship between the immune response and the development of severe malaria is essential for the management of the disease. There is currently insufficient data regarding immune parameters and relationship with severity of malaria infection in the study location. This study aims to determine the level of pro-inflammatory and anti-inflammatory cytokines as well as the immune cell ratios in children with malaria infection and ascertain their relationship with malaria severity.

## Materials and methods

### Study area

The study was conducted in Enugu, the capital of Enugu state in South-East Nigeria. The population is made up of mainly Igbo people who are civil servants and traders. The state lies partly within the semi-tropical rain forest belt of the south and is situated between *latitude* 06°21°N and *longitude* 07°26° E; and spreads towards the north through a land area of approximately 8727.1 km. The state’s total population consist of 4,411,119 inhabitants whereas the Enugu metropolis is about 840,000. The estimated prevalence of malaria parasitaemia in children aged 1 to 15 years in the geopolitical zone is 69.7% [9].

### Ethical approval

Prior to commencement of the study, ethical approval was obtained from the institutional review boards (IRB) of the University of Nigeria Teaching Hospital (UNTH), Ituku-Ozalla, Enugu (application reference no: NHREC/05/01/2008B-FWA00002458-IRB00002323 – UNTH/HREC/2023/06/530). A written informed consent was also obtained from each participant’s parent or caregiver before enrollment into the study.

### Study center

This study was carried out in the Paediatric clinics and Emergency ward of the University of Nigeria Teaching Hospital (UNTH), Ituku-Ozalla Enugu. The study period was from June 2023 to November 2023. The University of Nigeria Teaching Hospital is a federal tertiary health care institution which provides a wide range of a medical, surgical, diagnostic, out- patient, in-patient, rehabilitative and support services to its catchment population that includes all the states in South-East zone of Nigeria and other neighbouring states.

### Study design

A case-control design was adopted for the study. Participants were recruited consecutively as they come to the clinic or admitted into the ward until the estimated sample size was achieved. Other information required including the educational status of the child, both parent’s/caregiver’s highest educational attainment and occupation, and the presence of any malaria complications were retrieved from the participant’s case note.

### Study population

The study population consist of children aged six months to 12 years old admitted into the paediatric ward, out-patient paediatric clinics which hold daily at the hospital, and the children emergency ward of the hospital. There were three groups of participants: first group were children with complicated malaria; second group were children with uncomplicated malaria; and third group were healthy children who did not have malaria. The three groups were matched for age group, sex, and socioeconomic status of the parents. Information on the sociodemographic data of the participants was obtained from the parents or caregivers and in some cases, from children above 6 years. Blood transfusion history, comorbidities, and previous history of febrile illness were obtained from participants’ case notes.

### Case definitions

**Uncomplicated malaria** [10, 11] was defined as presence of fever with chills, sweats, headaches, nausea, vomiting, body aches, or general malaise, and the physical sign of pyrexia with tachypnoea, mild jaundice, splenomegaly, or hepatomegaly with presence of malaria parasite diagnosed by microscopy. There is absence of symptoms consistent with severe malaria.

**Complicated malaria** [10, 11] also known as severe malaria was defined as above but with associated abnormal behavior (depression/anxiety, sleep disorders, and aggressive behaviours), impairment of consciousness, seizures, coma, or other neurologic abnormalities (cerebral malaria); severe anaemia; acute respiratory distress syndrome (ARDS); coagulopathy, hypotension; acute kidney injury; hyperparasitemia, hypoglycaemia; metabolic acidosis; or hyper-parasitemia (more than 10% infected erythrocyte or more than 100,000 parasite per microliter of blood) diagnosed by microscopy. 

**Healthy controls** were recruited from apparently healthy children attending immunization clinic and in the general outpatient clinic for pre-school medical report of the hospital. They screened negative for malaria parasites by microscopy. These children also had no symptoms including fever (temperatures were within normal ranges), cough, vomiting, or any other symptoms of ill-health.

#### Socio-economic class

Using a composite index based on the average score for both parents’ highest educational attainment and occupation, the socioeconomic level of each child was ascertained [12, 13]. From 1 (highest) to 5 (lowest), there were five social classes [12]. Further divisions of the social classes were made into lower class (social classes 4 and 5) medium class (social class 3) and upper class (social classes 1 and 2).

#### Sample population

Using a complicated malaria rate of 5.5% obtained from previous study among children in the study area [14], at 95% confidence interval and 5% sampling error (precision), the calculated minimum sample size (n) was 71. However, considering a possible 10% attrition rate, 80 children were recruited for each of the three groups of the study.

#### Inclusion criteria

All children aged six months – 12 years accessing care in the hospital and diagnosed of malaria by microscopy were eligible for the study.

#### Exclusion criteria

Children with overt coexisting disease conditions such as upper and lower respiratory tract infections, septicaemia or meningitis were excluded. Children suspected to have septicaemia or meningitis (from history and or physical examination) were subjected to blood culture or cerebrospinal fluid analysis respectively and those with positive results were excluded from the study. Additionally, those children who have received a full course of artemisinin-based combination therapy (ACT) in the current illness or those that were on malarial prophylaxis, were also excluded from the study.

#### Laboratory analyses

A total of 2mls of venous blood was collected from the children less than 1 year old into ethylenediaminetetraacetic acid (EDTA) bottles. The full blood count (FBC), microscopy and malarial parasite density were done and afterwards, samples were centrifuged and the plasma was stored for estimation of IFN-γ, TNF-α and IL-10. For children aged above 1 year, 5mls of venous blood was collected from each participant. Two milliliters from the venous sample was dispensed into EDTA - anticoagulated bottle for the estimation of FBC, microscopy and malaria parasite density. The remaining 3 ml was dispensed into a clean and dry plain tube. This sample was allowed to stand for 30 min at room temperature in order to clot and retract and then centrifuged for 10 min at 3000 rpm. The serum was separated and used for the estimation of Serum IFN-γ, TNF-α and IL-10. Serum samples for cytokine analysis were batched, dispensed in aliquots using cryogenic vials. The samples were stored at -20 °C until analysis. Hematological parameters were measured within six hours of blood collection according to standard procedures.

### Laboratory procedure and analysis

#### Staining and examination of thin and thick smears

Four clean slides with frosted ends measuring 72 × 25 mm in dimension, 1 mm thick) were used. Two slides were used for thin blood film and the other two slides for the thick blood films. For the estimation of malaria parasite density, the *Plasmodium* parasites were counted against 200 WBC in thick film. However, 500 WBC were counted where less than 9 parasites were observed. Counting was done in the thin blood film against 2000 red blood cells. When counting was completed, the parasite density was calculated on the basis of the patient’s actual white cell count using the formula: Parasites / µL blood = Number of parasites counted x Actual white blood cells count divided by number of white blood cells counted [1, 15]. A parasite density of more than 250,000 per microliter was considered hyper-parasitaemia [1, 15].

#### Full blood count estimation

Venous blood was collected in ethylenediaminetetraacetic acid tubes. The tubes containing the anticoagulated blood were rocked gently on a multi-tube rotator. Samples were processed for full blood count parameters including hemoglobin level, packed cell volume, red cell indices, total white cell count with differentials and platelet count using the Mindray BC-2800 hematology autoanalyzer. The immune cell ratios – neutrophil lymphocyte ratio (NLR) and monocyte lymphocyte ratio (MLR) were calculated from the results of FBC using the formulae: NLR = neutrophils count x 10^9^/L /lymphocytes count x 10^9^/L and MLR = monocytes count x 10^9^/L/lymphocytes count 10^9^/L [16].

### Biochemical analysis - cytokine assay

The cytokines were measured using Elabscience™ human ELISA (Enzyme Linked ImmunoSorbent Assay) kit (Elabscience Biotechnology, Houston, TX, USA). It is a quantitative measurement of IFN-γ, TNF-α and IL -10 in serum, and cell-culture supernatant.

Briefly, 100 µl of standard and or sample was added to their respective wells and incubated at 37^O^C for 90 min. The liquid in each well was decanted and 100 µl of Biotinylated detection Ab/Ag, incubated at 37^0^C for 60 min. The plate was washed four times using Model RT-300 Rayto Life and Analytical Sciences Co., Ltd (China) automatic microplate washer set to dispense 350 µl wash buffer with soak time of one minute. Any fluid remaining in the wells was removed by tapping on clean paper absorbent towel. Then, 100 µl of the HRP conjugate working solution was added to each well, covered with another plate sealant and incubated for 30 min at 37^0^C. The plates were then washed for four times after which 90 µl of substrate reagent was added to each well, sealed with a new sealant, incubated in the dark for 20 min. Thereafter 50 µl of stop solution was added to each well. The plate was read with RT-2100 C Rayto Life and Analytical Sciences Co., Ltd (China) immunoassay microplate reader at 450 nm and four parameter logistic curve plotted with the Optical density (OD) on Y-axis and the concentration on X-axis. The OD of the samples was used to determine the respective concentration of the samples. Results were expressed in pg/ml.

The detection limits were as follows: 2 pg/ml for IL-10 and, 4 pg/ml for IFN-γ and TNF-α. All samples were tested in duplicates, and the mean of the two optical densities (OD) values was used for analyses.

#### Statistical analysis

Data was reported as mean ± standard deviation. Variables were compared between the three groups (complicated, uncomplicated, and healthy control) by using the non-parametric measures. The differences in mean values between the groups were analyzed for statistical significance using the analysis of variance (ANOVA) test. Possible correlations between levels of cytokines and immune cell ratios were identified using Pearson’s correlation coefficient. All statistical tests were done using the statistical packages for social sciences (SPSS) version 21.0 for Windows (SPSS Inc., Chicago IL, USA). A *P* values < 0.05 was considered statistically significant.

## Results

### Sociodemographic characteristics of the study participants

The overall mean age of the participants was 7.3 ± 3.4 (range 6 months − 12 years). Most of the participants, 155 (96.9%) were of the Igbo tribe and the male-female ratio was 1:1. Majority of the participants, 179 (74.6%) were between the 6–12 years age bracket, and over one third (36.3%) were in their secondary level of education. Seventy-five (31.3%)  of the participants’ parents/caregivers, were of low social class. The sociodemographic characteristics of the three groups including age, sex, educational level, and social class of their parents/caregivers were similar (*P* > 0.05). Details are as shown in Table 1.

**Table 1:** Sociodemographic characteristics of the study participants

Variable	subgroup	Group A	Group B	Control	F	*P*-value
		(*N*-80)	(*N*-80)	(*N*-80)		
**Age (Years)**	Mean age	6.85±3.65	7.63±3.36	7.45±3.28	1.13*	0.32
	<1	11(13.7%)	8(10%)	4(5%)		
	1-5	13(16.3%)	9(11.2%)	16(20%)		
	6-12	56(70%)	63(78.8%)	60(75%)		
**Sex**	Male	43(53.7%)	45(56.7%)	36(45%)	2.23^#^	0.33
	Female	37(46.3%)	35(43.3%)	44(55%)		
**Social Class**	Upper	27(33.7%)	25(31.3%)	26(32.5%)	0.39^#^	0.98
	Middle	29(36.3%)	28(35%)	30(37.5%)		
	Lower	24(30%)	27(33.7%)	24(30%)		
**Education**	None	11(13.7%)	15(18.7%)	14(17.5%)	1.16^#^	0.88
	Primary	28(35%)	29(36.3%)	30(37.5%)		
	Secondary	41(51.3%)	36(45%)	36(45%)		

*ANOVA (Analysis of variance); # = chi-square

### Levels of INF gamma, TNF alpha, and IL-10, among various groups

The mean levels of IFN- γ, in the complicated, uncomplicated and the healthy groups were 266.87 ± 66.34, 140.32 ± 30.38 and 32.90 ± 16.15 respectively (*P* < 0.001). Also, the mean cytokine levels were significantly higher in complicated than uncomplicated malaria (*p* < 0.001); and higher in uncomplicated malaria than normal healthy children (*p* < 0.001). Details are as shown in Table 2.

**Table 2:** The mean levels of the cytokines (INF gamma, TNF alpha, and IL-10) in children with complicated and uncomplicated malaria, and normal healthy children

Groups	IFN- gamma	TNF -alpha	IL-10
Complicated malaria (A) **(N = 80)**	266.87 ± 66.34	62.53 ± 6.37	40.60 ± 9.11
Uncomplicated malaria (B) **(N = 80)**	140.32 ± 30.38	42.02 ± 9.29	105.73 ± 32.06
Control (C) **(N = 80)**	32.90 ± 16.15	17.79 ± 6.03	18.74 ± 6.15
f-value*	322.140	418.944	249.787
*P*-value	< 0.001	< 0.001	< 0.001
A vs. B	< 0.001	< 0.001	< 0.001
A vs. C	< 0.001	< 0.001	< 0.001
B vs. C	< 0.001	< 0.001	< 0.001

INF-γ = interferon gamma; TNF-α = tumour necrotic factor alpha; IL-10 = Interleukin 10 *ANOVA

### The neutrophil-lymphocyte ratio and monocyte-lymphocyte ratio in children with complicated and uncomplicated malaria, and normal healthy children

The mean NLR levels in the complicated, uncomplicated and healthy control were 3.69 ± 3.99, 2.38 ± 1.53 and 1.29 ± 0.70 respectively (*P* < 0.001). The mean NLR was higher in children with complicated than uncomplicated malaria (*p* = 0.023); and normal healthy children (*p* < 0.001). However, there was no significant difference between the levels in uncomplicated and normal healthy children (*p* = 0.14). On the other hand, the mean MLR levels did not show any significant difference between the three groups: 0.13 ± 0.70, 0.08 ± 0.10 and 0.06 ± 0.04 respectively, *P* = 0.062). The details are as shown in Table 3.

**Table 3:** The neutrophil - lymphocyte ratio and monocyte - lymphocyte ratio in children with complicated and uncomplicated malaria, and normal healthy children*

GROUPS	NLR	MLR
Complicated malaria (A) **(N = 80)**	3.69 ± 3.99	0.13 ± 0.70
Uncomplicated malaria (B) **(N = 80)**	2.38 ± 1.53	0.08 ± 0.10
Control (C) **(N = 80)**	1.29 ± 0.70	0.06 ± 0.04
F-statistics*	10.053	2.835
P-value	< 0.001	0.062
A vs. B	0.023	0.132
A vs. C	< 0.001	0.203
B vs. C	0.141	1.000

NLR = Neutrophil lymphocyte ratio; MLR = Monocyte lymphocyte ratio; *ANOVA

### Levels of haemoglobin concentration, red cell indices, and platelets in children with complicated and uncomplicated malaria, and normal healthy children

The mean haemoglobin concentration (Hb), packed cell volume (PCV), and mean corpuscular haemoglobin (MCH), mean corpuscular haemoglobin concentration (MCHC), and platelets were significantly lower in children with complicated malaria than uncomplicated and control groups (*p* < 0.001). In contrast, the mean corpuscular volume (MCV) did not show any significant difference between the three groups of children. Details are as shown in Table 4.

**Table 4:** Levels of haemoglobin concentration, red cell indices, and platelets in children with complicated and uncomplicated malaria, and normal healthy children^*^

Study groups	Hb (g/dl)	PCV (L/L)	MCH (pg)	MCHC (g/dl)	MCV (fl.)	Platelet (x10^9^/L)
Complicated malaria (A) **(N = 80)**	7.8 ± 1.6	23.6 ± 5.1	27.5 ±3.	32.6 ± 2.1	78.7 ± 15.6	169.4 ± 56.4
Uncomplicated malaria (B) **(N = 80)**	10.1 ± 1.3	30.5 ± 5.0	28.9± 27	33.6 ± 1.2	83.6 ± 7.6	200.4 ± 60.6
Healthy children (C) **(N = 80)**	11.7 ± 1.8	36.3 ± 3.9	30.0 ± 28	35.1 ± 3.3	84.6 ± 13.9	294.8 ± 154.3
F-statistics	79.522	85.156	9.67	21.23	0.75	23.01
p-value	0.000	0.000	0.001	0.000	0.4735	0.000
A vs. B	0.000	0.000	0.0209	0.021	0.6811	0.159
A vs. C	0.000	0.000	0.001	0.000	0.4696	0.000
B vs. C	0.000	0.000	0.1429	0.024	0.9043	0.000

* = ANOVA; Hb = haemoglobin; PCV = packed cell volume; MCHC = mean corpuscular haemoglobin concentration; mean corpuscular volume

### Levels of total white blood cell counts and differentials in children with complicated and uncomplicated malaria, and normal healthy children

The mean total white blood cell (WBC) count was significantly higher in complicated than uncomplicated malaria group (*p* < 0.001); and higher in uncomplicated malaria group than healthy control (*p* = 0.01). The mean neutrophil and lymphocyte levels were significantly higher in complicated and uncomplicated malaria than healthy control (*p* < 0.001), but no difference between their mean levels in complicated and uncomplicated groups (*p* > 0.05). On the other hand, the mean level of monocyte is significantly higher in complicated than healthy control (*p* < 0.001), as well as in uncomplicated malaria (*p* = 0.026). Further details are as shown in Table 5.

**Table 5:** Levels of total white blood cell counts and differentials in children with complicated and uncomplicated malaria, and normal healthy children^*^

Study groups	WBC (x10^9^/L)	Neutrophil (%)	Lymphocyte (%)	Monocyte (%)	Eosinophil (%)
Complicated malaria (A) **(N = 80)**	11.2 ± 4.8	66.4 ± 15.9	30.1 ± 15.2	2.6 ± 1.7	1.2 ± 1.3
Uncomplicated malaria (B) **(N = 80)**	8.1 ± 4.4	62.9 ± 13.8	34.3 ± 13.7	1.8 ± 1.4	1.0 ± 1.3
Healthy Children (C) **(N = 80)**	5.6 ± 1.87	50.8 ± 12.9	45.6 ± 12.6	1.3 ± 1.4	1.1 ± 1.1
F-statistics	22.865	14.357	14.868	3.573	0.184
p-value	0.000	0.000	0.000	0.030	0.833
A vs. B	0.000	0.603	0.314	0.026	1.000
A vs. C	0.000	0.000	0.000	1.000	1.000
B vs. C	0.011	0.000	0.000	0.042	1.000

* = ANOVA; WBC = white blood cell

### Relationship between immune cell ratios (neutrophil lymphocyte ratio and monocyte lymphocyte ratio) and cytokines (interferon gamma, tumor necrotic factor, and interleukin-10) in children with malaria

There was a positive and significant correlation between neutrophil lymphocyte ratio [NLR] and interferon gamma [IFN-γ] {*r* = 0.815; *p* = 0.003}, as well as between NLR and TNF-α {*r* = 0.745; *p* = 0.002}. MLR also showed similar relationship (*r* = 0.831; *p* = 0.014 and *r* = 0.716; *p* = 0.001 respectively). However, NLR and MLR did not show any significant correlation with the IL-10 (*r* = -0.661; *p* = 0.642 and *r* = -0.115; *p* = 0.384 respectively).

## Discussion

This study has demonstrated that complicated (severe) malaria is associated with higher levels of pro-inflammatory cytokines (interferon-gamma [IFN-γ] and tumor necrotic factor-alpha [TNF-α]) and immune cell ratios (neutrophil lymphocyte ration [NLR] and monocyte lymphocyte ratio [MLR]), but lower level of anti-inflammatory cytokine (interleukin-10 [IL-10]) than uncomplicated malaria. The mean levels of haemoglobin concentration (Hb), packed cell volume (PCV), mean corpuscular hemoglobin (MCH) and mean corpuscular hemoglobin concentration (MCHC) were lower in complicated than uncomplicated malaria.

The immune cell ratio (NLR and MLR) levels correlated positively with pro-inflammatory cytokine (IFN-γ and TNF-α) levels but not with anti-inflammatory cytokine (IL-10).

The implications of these findings are that the pro-inflammatory cytokines are not only elevated in children with malaria as reported by previous authors [5, 17], but the elevation increases with disease severity. The increased elevation suggests an increased stimulation of phagocytosis to contain the increased parasitaemia often associated with complicated malaria [18, 19]. These observations are similar to the findings by previous authors [20–22]. For instance, the host cells elaborate IFN-γ, TNF-α, and IL-10 following exposure to malarial parasites and the increase in production of these cytokines contribute to the expression of the adhesion molecules often seen in patients with complicated and uncomplicated malaria. In acute phase of malaria infection pro-inflammatory cytokines such as TNF-α and IFN-γ are produced to eliminate the malaria parasites [23]. It is also a well known fact that overproduction of these cytokines can also lead to severe malaria with multiple end organ damage. Interleukin − 10 an anti-inflammatory cytokine is also elevated in the second phase of malarial infection [24]. The role is to inhibit the activities of the pro-inflammatory cytokines [24]. Tumour necrosis factor alpha plays a complex role by contributing to both immunopathology and protective immunity during malaria infection. Tumour necrosis factor alpha plays a crucial role in the progression of malaria to cerebral malaria and is also involved in anti-*Plasmodium* responses that result in intra-erythrocytic parasite killing and a reduction in parasitaemia when malaria is present [25]. Significant elevations in IFN-γ and TNF-α have been detected in tissue or blood in response to malaria infection. By promoting T-cell proliferation, producing reactive oxygen intermediates, and boosting macrophage phagocytic activity, these cytokines are known to assist in the removal of malaria parasites [25].

The burden of malaria is very high in Nigeria, and complicated malaria constitutes up to 11.3% of the cases in children [26]. It accounts for 12.4% of childhood mortality and 13.2% of childhood morbidity [27]. The overall effect of this burden is beyond health as it also adversely influences the developmental and economic indices of the country. All these indicate the need for more efforts on cutting-edge research that could improve the understanding and management of malaria in Nigeria and other malaria endemic countries of the world.

The observation that anti-inflammatory cytokine (IL-10) level is higher in children with uncomplicated than complicated malaria suggests that this cytokine may have a suppressive effect on the production of pro-inflammatory cytokines since its major role is to protect vital organs by limiting excessive inflammation [5, 28, 29]. It checks the production of pro-inflammatory cytokines while upregulating the expression of immunological checkpoint molecules on antigen-presenting cells and downregulating major histocompatibility complex-II and co-stimulatory molecule expression. The exciting finding of higher level of IL-10 suggests that this cytokine may be responsible for the less inflammation, less pathology, and better prognosis seen in children with uncomplicated malaria compared to complicated malaria. These children did not progress to severe disease likely because the inflammatory effects of Interferon-γ and TNF-α were well modulated by IL-10. This observation is similar to a report by Boeuf et al. 2012 [30], which showed a higher level of IL-10 in patients with uncomplicated malaria compared to complicated malaria. However, the findings from a recent systematic review and meta-analysis [31–33] were at variance with this finding, suggesting the need for further.

Haematological abnormalities can contribute to the pathogenesis and complications of malaria disease. The lower mean level of haemoglobin concentration observed in children with complicated malaria may be attributed to the marked phagocytosis and destruction of the parasitized erythrocytes seen with increasing severity [8]. Notable fluctuations have been seen in white blood cell count in malaria [34]. While some studies noticed leukopenia, others observed leucocytosis similar to what we found. The reason for the leukopenia was attributed to margination of the neutrophils especially in the spleen [35, 36]. The significantly higher level of total white blood cell count in complicated malaria may be due to the reduced immunity and possibly a higher parasitaemia seen in the complicated malaria group. According to several studies, leukocytosis has been observed in severe or complicated malaria and also in children. [37–39]. It is therefore not surprising to find a significantly higher neutrophil lymphocyte ratio in children with complicated malaria as observed in this study. The pattern of haematological indices seen in this study is consistent with those reported from malaria-endemic areas elsewhere [40, 41].

This study also showed positive and significant correlation between the immune cell ratios (neutrophil lymphocyte ratio and monocyte lymphocyte ratio) and the pro-inflammatory cytokines (IFN-γ and TNF-α). This finding suggests that the levels of immune cell ratios could be utilized in predicting the severity and prognosis of malaria disease. This will be beneficial in low- and middle- income countries (LMICs) where the pro-inflammatory biomarkers are extremely expensive and often inaccessible.

One of the major strengths of this study was the matching of the control groups to neutralize the effects of the notable cofounders such as age of the children and the socioeconomic status of the parents. Previous studies have shown that susceptibility to malaria infection increases after 6–9 months following waning of the maternal antibodies. Young children beyond this age bracket are more vulnerable than older children because of limited acquired immunity from previous exposures. Also, the matching for the socioeconomic status of the parents reduced the effect of social status vis a viz. diet and nutrition. Young children are most susceptible to malaria due to a lack of acquired functional immunity, which older children and adults develop due to multiple exposures.

### Study limitations

The short half-life of cytokines in plasma is a known drawback of utilizing immunoassays to quantify ex vivo cytokine levels. Therefore, these findings are viewed as minimum estimates of the concentrations of pro- and anti-inflammatory cytokines. Second, the blood samples for the estimates were all taken at a single point contact during the acute illness phase. Better results might be obtained by doing a longitudinal trial in which children who come with the two types of malaria are enrolled and then continuously monitored to obtain a time course curve for the cytokines. Although the children with overt coexisting disease conditions such as bronchopneumonia, septicaemia or meningitis were screened and excluded, it is possible some participants with covet conditions might have been included in the study. These infectious markers could affect the study outcomes; however, the estimated effect is expected to be minimal and non-directional because of the quality control groups. A random sample of eligible participants rather than a consecutive sample as used in this study would have given a more representative finding. Finally, the research findings of this study should be generalized with caution because of the limited number of cytokines analyzed in the study.

## Conclusion

Complicated malaria is associated with higher levels of pro-inflammatory cytokines and immune cell ratios. Additionally, the immune cell ratios levels correlate positively with pro-inflammatory cytokine, and could help in evaluation for the severity of malaria infection.

## Data Availability

The research data for this manuscript is available upon request to the corresponding author.
